# THE PREVALENCE AND PREDICTORS OF APATHY AMONG CANADIAN LONG-TERM CARE RESIDENTS: A SECONDARY DATA ANALYSIS

**DOI:** 10.1093/geroni/igad104.2299

**Published:** 2023-12-21

**Authors:** Aderonke Agboji, Shannon Freeman

**Affiliations:** University of Northern British Columbia, Prince George, British Columbia, Canada; University of Northern British Columbia, Prince George, British Columbia, Canada

## Abstract

Apathy, defined as a reduction in interest or motivation to engage in goal directed activities, is a frequent and persistent health concern among persons residing in long term care facilities. Research showed that apathy is associated with rapid cognitive decline, impaired functional ability, and increased mortality risk. To prevent or mitigate the negative impacts of apathy on persons living in long term care facilities, there is need for better understanding of the risk factors associated with it. Therefore, the aim of this study was to determine the prevalence of apathy and its risk factors in a large sample of Canadian long-term care residents (N=332,454). We performed cross-sectional analysis of data from MDS 2.0 assessments completed between 2015 and 2019 fiscal year. Results from descriptive and inferential analyses showed that apathy was more prevalent among persons aged 65 or younger and speak English. The strongest predictors of apathy were province of residence (OR 4.09 (CI 2.99-5.59) followed by resistance to care (OR 2.43 CI 2.38-2.49), conflict with family (OR 2.30 CI 2.2-2.4), and insomnia (OR 2.16 CI 2.11-2.2). These findings highlight the important role of environmental contextual factors including the physical and social environment as well as personal factors in the development of or recovery from apathy. Strategies for combating apathy in long term care facilities should take into account the individual, institutional and provincial variances.

